# Predicting in-hospital mortality in Iranian patients with spontaneous intracerebral hemorrhage

**Published:** 2014-10-06

**Authors:** Babak Bakhshayesh, Mozaffar Hosseininezhad, Seyed Mohammad Seyed Saadat, Morvarid Hajmanuchehri, Ehsan Kazemnezhad, Amir-Reza Ghayeghran

**Affiliations:** 1 Department of Neurology, School of Medicine, Poursina Hospital, Guilan University of Medical Sciences, Rasht, Iran; 2 School of Medicine, Guilan University of Medical Sciences, Rasht, Iran; 3 Guilan Road Trauma Research Center, Guilan University of Medical Sciences, Rasht, Iran; 4 Department of Biostatistics, School of Medicine, Guilan University of Medical Sciences, Rasht, Iran

**Keywords:** Intracerebral Hemorrhage, Outcome, Mortality, Hematoma

## Abstract

**Background: **Intracerebral hemorrhage (ICH) is the most fatal subtype of stroke. Despite limited effective therapy, there is no accepted clinical grading scale to predict in-hospital mortality, especially in developing nations. The purpose of this study was to assess the predictors of in-hospital mortality among a sample of Iranian patients with spontaneous ICH for use at the time of the first evaluation.

**Methods:** This prospective study was carried from January 2010 to the end of January 2011. Demographic, clinical, and laboratory data of ICH patients were collected. Hematoma volume and perihematoma edema (PHE) were measured on brain computed tomography scan using ABC/2 formula. Logistic regression analysis was performed to determine independent variables contributing to in-hospital mortality.

**Results: **Of a total 167 consecutive ICH patients, 98 patients met inclusion criteria. Mean ± standard deviation age of patients was 70.16 ± 12.52. After multivariate analysis, five variables remained as independent predictors of in-hospital mortality included: age [odds ratio (OR) = 1.12, 95% confidence interval (CI) = 1.03-1.23, P = 0.009], diabetes mellitus (OR = 10.86, 95% CI = 1.08-109.24, P = 0.009), National Institutes of Health Stroke Scale (NIHSS) score (OR = 1.41, 95% CI = 1.08-1.68, P ≤ 0.001), as well as volume of hematoma (OR = 1.1, 95% CI = 1.03-1.17, P = 0.003), and PHE (OR = 0.75, 95% CI = 0.60-0.93, P = 0.010).

**Conclusion: **Our results indicate that older age, diabetes mellitus, higher NIHSS, as well as larger volume of hematoma, and smaller PHE on admission are important predictors of in-hospital mortality in our ICH patients.

## Introduction

Spontaneous intracerebral hemorrhage (ICH) is the most fatal stroke subtype worldwide caused by spontaneous vascular rupture due to hypertension or amyloid angiopathy.^1^^-^^3^ The overall 30-days mortality rate for patient with intracranial hemorrhage is estimated to be 30-55%, of which about half die within the first 48-h.^4^^-^^7^ ICH is accounted for 10-20% of all strokes in the United States and between 20% and 30% in Asia.^8^

Predicting the risk of mortality following ICH is useful to determine prognosis, standardize the assessments and select the optimal treatment option.^9^^,^^10^ Moreover, accurate prediction of ICH outcome would assist both families and physicians to decide whether patients need to be transferred to an extended care facility.^11^ Although several grading scales have been proposed to predict mortality and outcome after ICH, an optimal predicting scale for in-hospital mortality that has been widely incorporated into clinical practice is lacking.^9^^,^^11^^-^^14^ The absence of such a standard grading scale has led to the inclusion of heterogeneous patients samples in randomized clinical trials and inconsistency in the clinical management of ICH patients.^9^^,^^10^

Although several studies on prognostic scales of ICH outcome have been conducted in western countries,^9^^,^^11^^-^^14^ fewer studies have evaluated the effect of prognostic indicators on in-hospital mortality. Moreover, few studies have attempted to investigate factors related to in-hospital mortality in developing countries where socioeconomic, geographic, and ethnic/racial characteristics are different.^15^^,^^16^ Therefore, this study was performed to assess the easily identifiable predictors of in-hospital mortality in a sample of Iranian patients with spontaneous ICH for use at the time of the first evaluation.

## Materials and Methods

This prospective study was conducted at Poursina Teaching-Hospital in Rasht, Iran, which serves as the main tertiary referral center for stroke in the province, from January 2010 to the end of January 2011. This study was approved by the Human Research Committee at the Guilan University of Medical Sciences (GUMS) (Rasht, Iran). All patients presented to the emergency department (ED) within the first 24 h of acute onset of focal neurological deficit whose brain Computed tomography (CT) scan on admission was compatible with a diagnosis of ICH were included in the study. Exclusion criteria were: (1) evidence of head trauma, (2) concomitant epidural or subdural hematoma, (3) history of stroke, bleeding tendency disorders, dementia, cancer, or any other severe concomitant illness, (4) secondary ICH (e.g., vascular malformations, aneurysm, tumor, trauma, vacuities, etc.), (5) neurosurgical intervention, and (6) transfer to another facility.

All patients were initially managed in the ED according to the guideline provided by American Heart Association/American Stroke Association for the initial management of cerebrovascular accident (CVA).^17^ A non-contrast spiral brain CT-scan was conducted within the first 6 h of admission using 7-10 mm slice thickness in the supratentorial regions and 5-mm slice thickness in the posterior fossa, scanned without gap. The aim of the study was explained to patients or their relatives, and written informed consent was obtained before participation. Demographic and clinical information, including age, sex, smoking, and history of hypertension (mmHg), diabetes mellitus, coronary artery disease, medication use, and other concomitant major illnesses were collected upon admission. Level of consciousness and severity of stroke was evaluated on arrival at the ED using Glasgow Coma Scale (GCS) and National Institutes of Health Stroke Scale (NIHSS), respectively. Two neurologists who were blinded to the radiological data collected patient’s demographic and clinical data. Baseline volumes of hematoma and perihematoma edema (PHE) volume, which were measured using ABC/2 method,^18^ location of ICH, presence of intraventricular hemorrhage (IVH) and subarachnoid extension of hematoma were recorded. The outcome was defined as in-hospital mortality. All images were reviewed by a neurologist and an expert radiologist who were blinded to the patient’s identity and clinical status. They measured the hematoma volume and PHE volume, and the consensus measurements were considered.

All statistical analyses were performed using SPSS for Windows 17.0 (SPSS Inc., Chicago, IL, USA). All independent variables used in the analysis of ICH outcome were chosen from medical literatures.^9^ The Kolmogorov–Smirnov test was applied to assess normality of data distribution. Chi-square test was used to compare categorical variables. Student’s t-test was used when data were normally distributed; otherwise, the Mann–Whitney U test was employed. The Spearman’s test was used to determine the correlation between hematoma volume and PHE. Variables with a P-value less than 0.100 in the univariate analysis were considered eligible for inclusion in the final multivariate model. Multiple logistic regression analysis using a backward likelihood ratio method was done to create a prediction outcome model. The goodness of fit of the model was evaluated by Hosmer and Lemeshow test. The level of significance was considered to be P < 0.050.

## Results

Of the total 167 consecutive ICH patients who admitted to the ED of our Hospital, 109 cases met inclusion criteria. Of these, 6 patients were excluded from the study because they lacked complete baseline data, 5 patients underwent neurosurgical intervention, 21 presented to the ED with delay more than 24 h; 12 had concomitant major abnormal findings other than ICH on brain CT-scan and 14 had a positive history of diseases consistent with the exclusion criteria. In addition, 9 patients were excluded during the in-hospital follow-up period, of which 5 were diagnosed as having secondary ICH, 2 had radiologic evidence of head trauma, and 3 were discharged against medical advice and continue their treatment in a private hospital. Finally, the remaining 98 patients constituted the population of our study. All patients were treated conservatively. Conservative management included airway protection, stabilization of vital signs, control of hypertension, treatment of complications like increased intracranial pressure.

The demographic and clinical characteristics, as well as radiologic findings of the patients and the association of each variable with in-hospital mortality, are given in table 1. Mean age of patients was 70.16 ± 12.52 years, the average time between the onset of symptoms and admission was 7.05 ± 4.75 h, mean GCS was 11.00 ± 4.04, and mean NIHSS score was 16.44 ± 9.75. Male to female ratio was 1.08/1.00. The prevalence of diabetes mellitus, hypertension, and coronary artery disease among ICH patients were 27.6%, 63.3% and 21.4% respectively. IVH was seen in 42.9% of patients. Mean hematoma volume and mean volume of PHE were 29.38 ± 23.09 and 23.29 ± 7.60 cc, respectively. Most frequent locations of hematoma were thalamus (34.7%), basal ganglia (32.7%), cerebral hemispheres (20.4%), cerebellum (8.2%), and brainstem (4.1%). The overall mortality rate in this study was 30.6% in the hospital. Of these, 40.0% occurred in the first 2 days of hospitalization.

On univariate analysis, deceased patients were older (P < 0.001) and had significantly lower GCS (P < 0.001), higher NIHSS score (P < 0.001), and larger PHE (P = 0.006) and hematoma volume (P < 0.001) than those who survived. Moreover, diabetes mellitus [OR = 2.9, 95% confidence interval (CI) = 1.16-7.48, P = 0.020], IVH (OR = 3.16, 95% CI = 1.29-7.74, P = 0.010), and location of hematoma (P = 0.001) were significantly associated with increased risk of in-hospital mortality (Table 1). Spearman’s rank test showed that hematoma volume was significantly correlated with PHE (P < 0.001, r = 0.736). The results of multivariate analysis using a logistic regression model are summarized in table 2. The whole model was significant (P < 0.001) and the overall accuracy of the model was 92.9%. The Hosmer and Lemeshow test demonstrated a very good fit of the model (P < 0.999). Presence of IVH, and GCS no longer remained significant, after adjustment for the other confounding variables. Furthermore, location of the hematoma was not associated with increased risk of mortality, even after exclusion of four patients with brainstem hemorrhage and redefinition of the supratentorial hemorrhages as both hemispheric and thalamic hemorrhages in reanalysis. After adjustment for potential confounding factors, five variables remained as significant predictors of in-hospital mortality: diabetes mellitus (OR = 10.86, 95% CI = 1.08-109.24, P = 0.009), NIHSS score (OR = 1.41, 95% CI = 1.08-1.68, P ≤ 0.001), volume of hematoma (OR = 1.10, 95% CI = 1.03-1.17, P = 0.003), PHE (OR = 0.75, 95% CI = 0.60-0.93, P = 0.010), and age (OR = 1.12, 95% CI = 1.03-1.23, P = 0.009). 

**Table 1:** Univariate analysis of the association between demographic and clinical characteristics as well as radiologic findings of patients with intracerebral hemorrhage with in-hospital mortality

**Variables**	**All (n = 98)**	**Survivors ** **(n = 68)**	**Non-survivors ** **(n = 30)**	**P**
Age (year)	70.16 ± 12.52	67.10 ± 11.07	77.10 ± 13.02	< 0.001
Gender				
Male, n (%)	53 (54.1)	37 (69.8%)	16 (30.2%)	0.921
Female, n (%)	45 (45.9)	31 (68.9)	14 (31.1)	
History of CAD				
Yes, n (%)	21 (21.4)	13 (61.9)	8 (38.1)	0.401
No, n (%)	77 (78.6)	55 (71.4)	22 (28.6)	
History of HTN				
Yes, n (%)	62 (63.3)	44 (71.0)	18 (29.0)	0.656
No, n (%)	38 (36.7)	24 (66.7)	12 (33.3)	
History of DM				
Yes, n (%)	27 (27.6)	14 (51.9)	13 (48.1)	0.020
No, n (%)	71 (72.4)	54 (76.1)	17 (23.9)	
Time elapsed from symptom onset to admission (h)	7.05 ± 4.75	7.38 ± 5.31	6.30 ± 3.12	0.211
Presence of IVH				
Yes, n (%)	43 (43.9)	24 (55.8)	19 (44.2)	0.01
No, n (%)	55 (56.1)	44 (80.0)	11 (20.0)	
GCS	11.00 ± 4.04	12.82 ± 2.36	6.87 ± 4.02	< 0.001
NIHSS	16.44 ± 9.75	11.91 ± 6.32	26.70 ± 8.27	< 0.001
Hematoma volume (cc)	29.38 ± 23.09	20.31 ± 13.31	49.93 ± 27.28	< 0.001
Volume of PHE (cc)	23.29 ± 7.61	21.51 ± 5.79	27.3 ± 9.61	0.006
Location of hematoma				
Thalamus, n (%)	34 (34.7)	23 (67.6)	11 (32.4)	0.001
Basal ganglia, n (%)	32 (32.7)	29 (90.6)	3 (9.4)	
Lobar, n (%)	20 (20.4)	10 (50.0%)	10 (50.0)	
Cerebellar, n (%)	8 (8.2)	6 (75.0)	2 (25.0)	
Brainstem, n (%)	4 (4.1)	0 (0.0)	4 (100.0)	

CAD: Coronary artery disease; HTN: Hypertension (mmHg); DM: Diabetes mellitus; IVH: Intraventricular hemorrhage

PHE: Perihematoma edema; GCS: Glasgow Coma Scale; NIHSS: National Institutes of Health Stroke Scale

**Table 2:** Results of multivariate analysis of predictors of in-hospital mortality

**Variables**	**OR**	**95% CI**	**P**
Age	1.12	1.03-1.23	0.009
DM	10.86	1.08-109.24	0.043
NIHSS	1.41	1.18-1.68	< 0.001
Hematoma volume	1.10	1.03-1.17	0.003
PHE	0.75	0.6-0.93	0.010

DM: Diabetes mellitus; NIHSS: National Institutes of Health Stroke Scale; PHE: Perihematoma edema, CI: Confidence interval

OR: Odd ratio

As, patients with diabetes mellitus were 10.86 times more likely to die compared to those without diabetes mellitus and each 10-year increase in age increased the odd of death by 3.3 folds. Moreover, for every 10 score increases in NIHSS, the risk of death increased nearly 32 folds. Finally, each 10 cc increase in hematoma volume increased the rate of death 2.7 fold, while for every 10 cc decline in PHE the risk of death increased by 16.7 folds.

## Discussion

Developing a standard clinical grading scale has an essential role in triage, assessment, and treatment of patients with ICH and designing clinical trials.^9^ Until date, numerous clinical studies have paid attention to determine the prognostic factors of outcome after ICH and have proposed several grading scales in different populations.^9^^,^^11^^-^^14^^,^^19^ However, few studies have attempted to investigate factors related to in-hospital mortality, especially in developing countries. The goal of our study was to investigate independent prognostic factors of in-hospital mortality in Iranian patients with spontaneous ICH for use at the time of the first evaluation in the ED. In contrast to most previous studies,^11^^-^^14^ which did not consider the time of CT scan, we only included patients who presented to our hospital within 24 h of symptoms onset whose CT scan was performed within 6 hours of admission. It is, therefore, likely that our results would generalize to more typical cases of ICH.

We found that older age, diabetes mellitus, neurological impairment according to NIHSS score, larger hematoma volume and smaller PHE at admission are five important predictors of in-hospital mortality in our patients with ICH. However, we did not identify any association between the in-hospital mortality and other variables, including history of hypertension, gender, and location of hemorrhage. Even after exclusion of brainstem hemorrhages and redefinition of the supratentorial hemorrhages as hemispheric and thalamic hemorrhages, location of the hematoma was not remained significant in the final regression model.

Previous studies have proposed several predictors for mortality of ICH patients.^12^^,^^14^^-^^16^ Consistent with the most previous studies,^12^^,^^14^^-^^16^ older age was the important predictor of in-hospital mortality in our study both on univariate and multivariate analysis. The results of the current study also support a finding by others^15^^,^^20^ that diabetes mellitus is independently associated with high mortality rate of ICH patients, albeit with a rather wide CI, this association needs to be interpreted with caution. The result of our study supports the finding of previous studies,^21^^,^^22^ that greater neurological severity, as assessed by NIHSS, is associated with poor outcome. However, in contrary to the findings of Ruiz-Sandoval et al.^14^ GCS was not associated with in-hospital mortality in our study, when adjusted for other potential variables. The advantage of NIHSS over GCS in predicting outcome following ICH could be explained by the fact that NIHSS not only has a wider spectrum than GCS to assess neurological dysfunction but also is able to measures the level of consciousness.^23^^,^^24^ However, GCS which is a reliable tool for assessment of severity and level of consciousness, especially following traumatic brain injury, is not an indicator of the whole neurological status.^21^^,^^22^

This study confirmed previous research findings^12^^,^^14^^,^^15^^,^^25^ indicating that increased hematoma volume is associated with mortality of ICH patients. However, contrary to most prior studies,^10^^,^^14^^,^^15^ IVH was not an independent predictor of mortality, when adjusted for other factors. Prognostic significance of early PHE on clinical outcome of ICH patients is controversial in the literature.^26^^-^^28^ Arima et al.^27^ showed that neither absolute nor relative increase in PHE volume during the first 72 h was associated with death or 90-day functional outcome, when adjusted for age, sex randomized treatment and baseline hematoma volume. Surprisingly, although PHE was directly associated with in-hospital mortality on univariate analysis, we found that decreased PHE volume is significantly associated with higher in-hospital mortality of ICH patients on multivariate analysis. This difference might reflect the confounding influence of other variables. Similar to previous studies,^27^^-^^29^ PHE was significantly correlated to baseline hematoma volume. This indicates that larger hematoma volume causes larger PHE. Similar to most previous studies, we only evaluated a snapshot of patient's clinical conditions and did not assess the dynamic evolution of characteristics, such as hematoma expansion and PHE growth over time, and their effects on the final outcome measures. Further investigations to evaluate the prognostic significance of hematoma expansion and PHE growth are recommended.

This study had some limitations: (1) Patients with secondary ICH and pre-existing disability were excluded. So we cannot generalize our results to all ICH patients. (2) In contrary to most previous studies, we considered in-hospital mortality as our outcome measure to evaluate the function of the hospital stroke team and to eliminate the potential effects of socioeconomic status, as well as other patient and family-related factors on later clinical outcomes. (3) Because our study performed in a teaching hospital serves as a referral tertiary center of stroke patients, we might have selected patients with lower socioeconomic status at presentation and also during the follow-up periods, as we lost three patients who decided to leave our hospital to continue treatment in another private hospital. Therefore, potential referral and selection bias are the third limitation of this study. (4) The lack of data on some other factors such as the use of anti-platelets and anti-coagulants, baseline laboratory values, as well as CT evidence of midline shift, hydrocephalus, and herniation, which may restrict the generalizability of our findings. (5) Although the use of ABC/2 method has been validated for the estimation of ICH volume, recent studies showed that this technique is not accurate enough in measuring the volume of hematoma and PHE. (6) Our relatively small sample size, which may have limited our statistical power to detect all of the variables associated with in-hospital mortality.

## Conclusion

Our results indicate that age, diabetes mellitus, NIHSS, and volume of hematoma, and PHE can predict the risk of in-hospital mortality on presentation in patients with spontaneous ICH.

## Notes

**How to cite this article: **Bakhshayesh B, Hosseininezhad M, Seyed Saadat SM, Hajmanuchehri M, Kazemnezhad E, Ghayeghran AR. Predicting in-hospital mortality in Iranian patients with spontaneous intracerebral hemorrhage. Iran J Neurol 2014; 13(4): 231-6.
